# A novel prognostic nomogram predicts premature failure of kidney allografts with IgA nephropathy recurrence

**DOI:** 10.1093/ndt/gfad097

**Published:** 2023-05-18

**Authors:** Kamila Bednarova, Geir Mjøen, Petra Hruba, Istvan Modos, Ludek Voska, Marek Kollar, Ondrej Viklicky

**Affiliations:** Department of Nephrology, Transplant Centre, Institute for Clinical and Experimental Medicine, Prague, Czech Republic; 1st Medical Faculty, Charles University, Prague, Czech Republic; Department of Nephrology, Oslo University Hospital, Oslo, Norway; Transplant Laboratory, Institute for Clinical and Experimental Medicine, Prague, Czech Republic; Department of Informatics, Institute for Clinical and Experimental Medicine, Prague, Czech Republic; Clinical and Transplant Pathology Centre, Institute for Clinical and Experimental Medicine, Prague, Czech Republic; Clinical and Transplant Pathology Centre, Institute for Clinical and Experimental Medicine, Prague, Czech Republic; Department of Nephrology, Transplant Centre, Institute for Clinical and Experimental Medicine, Prague, Czech Republic; Transplant Laboratory, Institute for Clinical and Experimental Medicine, Prague, Czech Republic

**Keywords:** glomerulonephritis, IgA nephropathy, kidney transplantation, nomogram, recurrence

## Abstract

**Background:**

Recurrence of immunoglobulin A nephropathy (IgAN) limits graft survival in kidney transplantation. However, predictors of a worse outcome are poorly understood.

**Methods:**

Among 442 kidney transplant recipients (KTRs) with IgAN, 83 (18.8%) KTRs exhibited biopsy-proven IgAN recurrence between 1994 and 2020 and were enrolled in the derivation cohort. A multivariable Cox model predicting allograft loss based on clinical data at the biopsy and a web-based nomogram were developed. The nomogram was externally validated using an independent cohort (*n* = 67).

**Results:**

Patient age <43 years {hazard ratio [HR] 2.20 [95% confidence interval (CI) 1.41–3.43], *P* < .001}, female gender [HR 1.72 (95% CI 1.07–2.76), *P* = .026] and retransplantation status [HR 1.98 (95% CI 1.13–3.36), *P* = .016] were identified as independent risk factors for IgAN recurrence. Patient age <43 years [HR 2.77 (95% CI 1.17–6.56), *P* = .02], proteinuria >1 g/24 hours [HR 3.12 (95% CI 1.40–6.91), *P* = .005] and C4d positivity [HR 2.93 (95% CI 1.26–6.83), *P* = .013] were found to be associated with graft loss in patients with IgAN recurrence. A nomogram predicting graft loss was constructed based on clinical and histological variables, with a C statistic of 0.736 for the derivation cohort and 0.807 for the external validation cohort.

**Conclusions:**

The established nomogram identified patients with recurrent IgAN at risk for premature graft loss with good predictive performance.

## Graphical Abstract

**Graphical Abstract:**
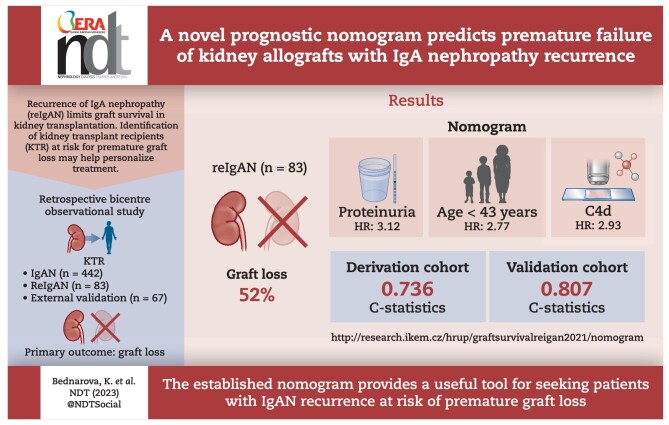

KEY LEARNING POINTS **What was known:**

Recurrence of immunoglobulin A nephropathy (IgAN) is associated with an increased risk of allograft loss, but predictors of poor outcomes are poorly understood.

**This study adds:**

A prognostic web-based nomogram based on clinical variables at biopsy (patient age, proteinuria, C4d staining, antihypertensive treatment, estimated glomerular filtration rate and time to recurrence) accurately predicts premature allograft loss.

**Potential impact:**

Future innovative interventions may help to slow down the progression of recurrent IgAN among identified patients at risk.

## INTRODUCTION

The recurrence of immunoglobulin A nephropathy (IgAN) is a common complication in kidney transplantation, significantly affecting the long-term graft outcomes to a similar extent as chronic antibody-mediated rejection [1–4]. The reported incidence varies between 9 and 53% according to different studies, with higher rates in centres performing protocol biopsies [1, 5, 6]. Despite an excellent outcome early after transplantation, almost 50% of recurrent patients experience premature allograft loss [1]. Identifying high-risk patients has been challenging, as the risk of progression to allograft failure is heterogeneous.

Many clinical factors determine the risk of progression of the original disease, including hypertension, young age, proteinuria, the Oxford MEST-C score, low estimated glomerular filtration rate (eGFR), presence of C4d and crescents in kidney biopsy [7–18]. Similarly, several studies have shown associations of IgAN recurrence with young age at transplant, rapid progression of the native disease, retransplantation, pre-emptive transplants, living donors and early steroid withdrawal [1, 2, 4–6, 19–23]. However, most of the performed studies have focused mainly on the risk factors for IgAN recurrence. The identification of risks associated with premature graft loss in a case of recurrent disease is an unmet medical need in kidney transplantation, and prediction models are not available.

In this study we aimed to identify risk factors associated with biopsy-proven IgAN recurrence and subsequent graft loss in the well-identified patient cohort and construct a prognostic nomogram identifying patients at risk for premature graft loss.

## MATERIALS AND METHODS

### Patients

**Figure 1:**
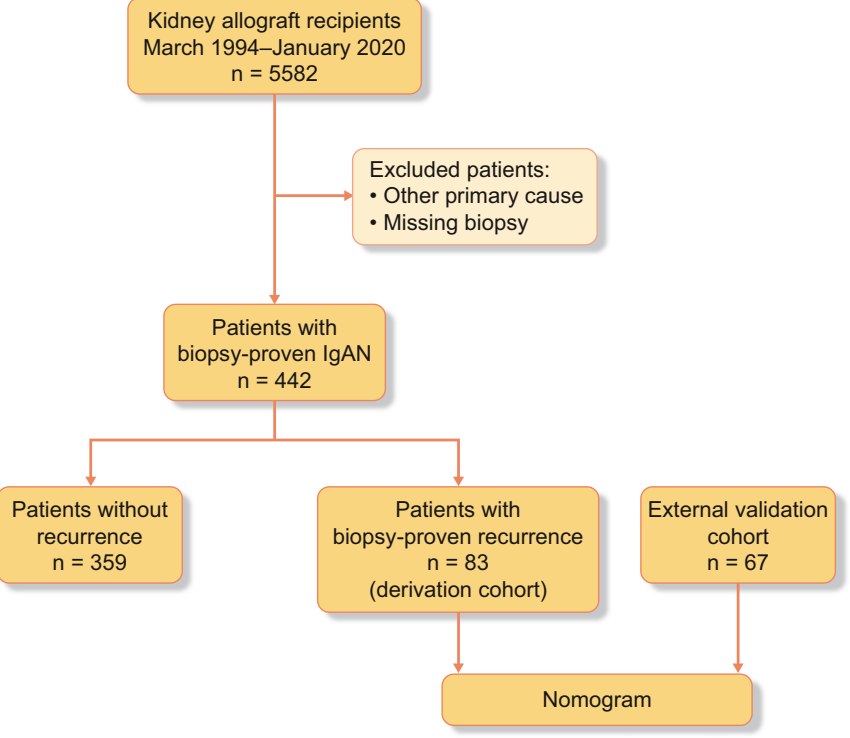
IgAN.

In this retrospective observational cohort study, 442 of 5582 patients had undergone kidney transplantation due to biopsy-proven IgAN from 1994 to 2020 (Fig. 1). Most patients were males who received primary kidney transplants from deceased donors. Repeat transplantations were performed in 66 patients. Detailed patient and donor characteristics are given in Table 1. The recurrence of IgAN after kidney transplantation was biopsy proven by case or protocol biopsies in 83 patients (18.8%) during the study follow-up. The first biopsy, which proved the IgAN recurrence, was used as a reference for laboratory and clinical assessments.

**Table 1:** Patient demographics.

		Biopsy-verified IgAN recurrence		
Characteristics	Entire cohort (*N* = 442)	Yes (*n* = 83)	No (*n* = 359)	*P*-value^a^
Recipient age (years), median (min–max)	45 (18–79)	40 (18–63)	46 (19–79)	<.0001
Recipient gender (male), *n* (%)	347 (79)	58 (70)	289 (80.5)	.038
Donor age (years), median (min–max)	51 (1–81)	51 (19–76)	51 (1–81)	.694
Donor gender (male), *n* (%)	135 (30.5)	14 (16.8)	121 (33.7)	.140
Retransplantation, *n* (%)	66 (14.9)	21 (25.3)	45 (12.5)	.003
Living donor, *n* (%)	117 (26.5)	24 (28.9)	93 (25.9)	.575
Peak PRA, median (min–max)	4 (0–98)	8 (0–96)	4 (0–98)	0.066
HLA mismatch, median (min–max)	3 (0–6)	3 (0–6)	3 (0–6)	0.852
Dialysis vintage (months), median (min–max)	18 (0–129)	16 (0–83)	18 (0–129)	0.841
Cold ischaemia (hours), median (min–max)	14 (0–27)	14 (0–26)	14 (0–27)	0.612
Post-transplant, *n* (%)				
Repeated erythrocyturia	120 (27.1)	51 (61.4)	69 (19.2)	<0.001
Repeated proteinuria	140 (31.7)	56 (67.5)	84 (23.4)	<0.001
T-cell-depletive induction	165 (37)	36 (44)	129 (36)	0.165

Erythrocyturia was defined as >10 erythrocytes/µl. Proteinuria was defined as urinary protein excretion >0.3 g/day.

^a^ *P*-values for group comparison based on the Mann–Whitney U test for continuous variables and Pearson's chi-squared test for categorical variables. *P* < .05 was considered significant.

To study risk factors for IgAN recurrence, the following variables were recorded: gender, age, donor type, donor age, human leucocyte antigen (HLA) mismatch, panel reactive antibody (PRA), cold ischaemia time, time on dialysis, induction immunosuppression, any proteinuria, erythrocyturia and date of graft loss or patient death.

To study risk factors for graft loss, variables obtained at the date of graft biopsy-confirming recurrence were eGFR, proteinuria (>1 g/day), erythrocyturia (>10 erythrocytes/µl), C4d staining in histology, time to recurrence, MEST-C score, antihypertensive therapy, renin–angiotensin–aldosterone system (RAAS) inhibitors, steroid therapy and indication biopsy status.

The maintenance immunosuppression consisted of calcineurin inhibitors [CNIs; *n* = 78 (94%); 9 on cyclosporine, 69 on tacrolimus] in combination with mycophenolate mofetil [MMF; *n* = 71 (91%)] and steroids [*n* = 77 (93%)]. Five patients had received CNI-free regimens. Because the vast majority (95%) of patients had received tacrolimus along with MMF for immunosuppression, those variables were not particularly studied. In patients with histologically proven IgAN recurrence, steroid pulses were initiated in cases with present crescents (*n* = 4), RAAS inhibitors were given to all patients with recurrent IgAN.

The patient clinical and histological data were retrieved from the hospital's internal database. This article is compliant with the Strengthening the Reporting of Observational Studies in Epidemiology statement.

### Pathology

A total of 83 graft biopsies confirming recurrence were scored according to the Oxford MEST-C scoring system [17], defined as follows: mesangial hypercellularity ≤0.5 (M0) and >0.5 (M1); endocapillary hypercellularity absence (E0) and presence (E1); segmental glomerulosclerosis absence (S0) and presence (S1); tubular atrophy/interstitial fibrosis <25% (T0), 25–50% (T1) or >50% (T2); and cellular/fibrocellular crescents absent (C0), 1–25% (C1) or >25% (C2).

### Validation cohort

An independent cohort of 67 patients transplanted between 1979 and 2020 from Oslo University Hospital was included in the study as a validation cohort to validate nomogram performance. The demographics of the validation cohort are given in Supplementary Table S1.

### Statistics

Data are presented as medians with minimum and maximum for continuous variables and counts with percentages for categorical variables. Continuous variables were compared by the Mann–Whitney U test and categorical variables by Pearson's chi-squared test. The graft and patient survival, proteinuria and erythrocyturia-free intervals were evaluated by Kaplan–Meier analysis using the logrank test. To investigate the significance of each prognostic factor for IgAN recurrence and allograft loss, univariable Cox proportional hazards models were created. The optimal cut-off point for recipient age as a risk for IgAN recurrence was defined by receiver operating characteristics (ROC) analysis at the maximal specificity and sensitivity (as <43 years of age). Potential predictors of graft loss/IgAN recurrence were included in the multivariable model based on their significance in the univariable analysis after checking their co-linearity. Patients with missing values in the evaluated variables were excluded from the analysis.

To identify prognostic predictors (known at the time of diagnostic biopsy) of graft loss in patients with IgAN recurrence, we developed a multivariable Cox regression model based on the most significant variables from the univariable model (*P* < .2) and clinically relevant parameters. Multicollinearity of explanatory variables was checked by calculation of the variance inflation factor (VIF) using the car package in R software (https://CRAN.R-project.org/package=car) and variables with the highest VIF were not retained in the final model (MEST-C score and indication biopsy status). A nomogram was constructed to predict 2- and 5-year prognosis after diagnostic biopsy with IgAN recurrence as previously described, using the rms package in R software (https://CRAN.R-project.org/package=rms) [24]. The DynNom package (https://CRAN.R-project.org/package=DynNom) was used to create a dynamic nomogram, which was then deployed on https://www.shinyapps.io/. The dynamic nomogram is publicly accessible at http://research.ikem.cz/hrup/graftsurvivalreigan2021/nomogram.

The internal validation of the nomogram was performed with the bootstrap set to 1000. The discrimination of the nomogram was evaluated by a concordance index and a calibration plot was performed to evaluate the prediction performance of the nomogram.

An independent cohort of 67 patients with IgAN recurrence from Oslo University Hospital was used for external validation. The discrimination of nomograms in the derivation and external cohorts was evaluated by the concordance index, which was internally validated with a bootstrap set to 1000. The calibration plots were performed to evaluate the prediction performance of the nomogram by comparing the observed and predicted survival probability over the 2 and 5 years after IgAN recurrence in derivation and external cohorts using the rms package in R software.

*P*-values <.05 were considered significant. Statistical analysis was performed using R-studio version 4.0.3 (2020.10.10).

## RESULTS

### Recurrence of IgAN

**Figure 2:**
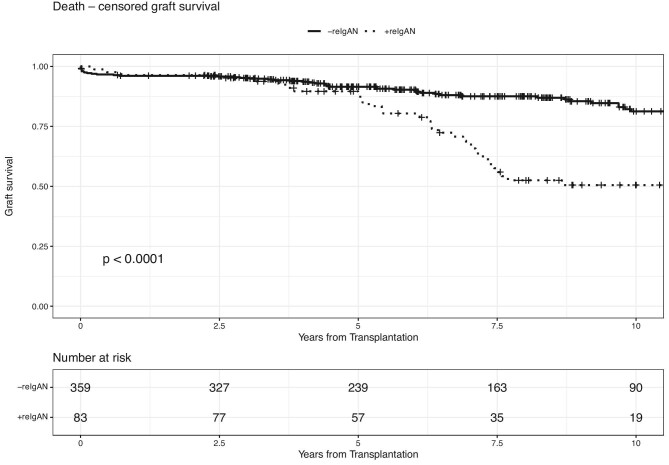
Patients with recurrence of the native disease have the same 5-year (88.1% versus 91.5%, logrank *P* = .4) and worse 10-year (50.5% versus 88.1%, logrank *P* < .0001) allograft survival compared with those without recurrence.

The recurrence of IgAN was reported in 83 of 442 patients with biopsy-proven native kidney IgAN, with a median time to recurrence of 2.3 years. Those who experienced disease recurrence were likely to be younger at transplant compared with those without recurrence (median 40 versus 46 years; *P* < .0001). Similarly, more patients with IgAN recurrence had undergone retransplantation (25.3% versus 12.5%; *P* = .003) (Table 1). Proteinuria and haematuria were more common in patients with IgAN recurrence as compared with those without proven recurrence (67.5% versus 23.4%; *P* < .001 and 61.4% versus 19.2%; *P* < .001, respectively). C4d staining was detected in 14 samples. In all but three of them, glomerular deposits were present to various extents. Recipients with disease recurrence displayed significantly worse 10-year graft survival compared with recipients without recurrence (logrank *P* < .001) (Fig. 2).

### Risk for IgAN recurrence

A Cox proportional hazards regression was applied to generate the proposed risk model of IgAN recurrence based on factors known at transplantation. Four statistically significant predictors of recurrent IgAN in the allograft were revealed in the univariable analysis: younger age at the time of transplantation [HR 2.14 for patients <43 years of age (95% CI 1.38–3.36), *P* < .001], retransplantation [HR 2.34 (95% CI 1.41–3.88), *P* < .001], higher peak PRA [HR 1.01 (95% CI 1.00–1.02), *P* = .02] and female gender of the recipient [HR 1.75 (95% CI 1.09–2.81), *P* = .019]. Only recipients <43 years of age [HR 2.20 (95% CI 1.41–3.43), *P* < .001], female gender of the recipient [HR 1.72 (95% CI 1.07–2.76), *P* = .026] and retransplantation [HR 1.98 (95% CI 1.13–3.36), *P* = .016] remained significant variables in multivariable analysis. A Cox proportional hazards model was created including patients after the first transplantation only. Similarly, younger age and female gender remained significant in multivariable analysis after the exclusion of retransplanted patients (*P* < .001 and *P* = .016, respectively) (Table 2, Supplementary Table S2).

**Table 2:** Variables associated with recurrent IgAN.

Predictors	Univariable analysis,^a^ HR (95% CI)	*P*-value	Multivariable analysis,^a^ HR (95% CI)	*P*-value
**Total cohort**				
Recipient age <43 years	2.14 (1.38–3.36)	<.001	2.20 (1.41–3.43)	.001
Retransplantation	2.34 (1.41–3.88)	.001	1.98 (1.13–3.36)	.016
Peak PRA^b^	1.01 (1.00–1.02)	.020	1.01 (0.99–1.01)	.169
HLA mismatch	1.01 (0.88–1.17)	.856	–	–
Recipient gender (female)	1.75 (1.09–2.81)	.019	1.72 (1.07–2.76)	.026
Dialysis vintage (months)	1.00 (0.99–1.01)	.785	–	–
Cold ischaemia (hours)	0.99 (0.98–1.01)	.387	–	–
Donor age (years)	1.01 (1.00–1.03)	.136	–	–
Donor gender (male)	0.65 (0.42–1.02)	.062	–	–
Living donor	1.19 (0.74–1.91)	.481	–	–

^a^Univariable and multivariable associations were calculated by Cox regression. *P* < .05 was considered significant.

^b^PRA measurement was not available in one patient.

^c^HLA mismatch not available in 29 patients.

^d^Dialysis vintage unknown in 45 patients.

^e^Cold ischaemia unknown in 53 patients.

^f^Donor age and gender unknown in 21 and 20 patients, respectively.

### Outcomes of recurrent IgAN

Patients with biopsy-proven IgAN recurrence experienced worse 10-year graft survival (logrank *P* < .001) compared with those without proven recurrence (Fig. 2). In 43 of the 83 patients with IgAN recurrence, the allograft loss was noticed at a median of 3.2 years (minimum 0.2, maximum 12) after the graft biopsy. The median time from transplantation to graft loss was 6.8 years.

In univariable Cox regression analysis, age <43 years [HR 3.76 (95% CI 1.72–8.20), *P* = .001], erythrocyturia [HR 2.49 (95% CI 1.32–4.69), *P* = .005], proteinuria >1 g/day [HR 3.19 (95% CI 1.64–6.23), *P* = .001], indication biopsy [HR 3.76 (95% CI 1.56–9.06), *P* = .003] and C4d-positive staining in the allograft biopsy [HR 2.96 (95% CI 1.43–6.11), *P* = .003] were found to be associated with graft loss. From the MEST-C score, the T lesion [HR 1.98 (95% CI 1.26–3.11), *P* = .003] and the sum of MEST-C scores [HR 1.25 (95% CI 1.02–1.53), *P* = .036] significantly predicted graft loss (Table 3).

**Table 3:** Variables associated with graft failure in biopsy-proven recurrent IgAN.

	Univariable analysis^a^		Multivariable analysis^a^	
Predictors	HR (95% CI)	*P*-value	HR (95% CI)	*P*-value
Recipient age <43 years	3.76 (1.72–8.20)	.001	2.77 (1.17–6.56)	.020
Time to recurrence (years)	1.07 (1.00–1.15)	.051	1.03 (0.96–1.12)	.423
eGFR at biopsy with recurrence (ml/s/1.73 m^2^)	0.43 (0.15–1.23)	.117	0.62 (0.2–1.95)	.415
Erythrocyturia >10/µl at the time of biopsy	2.49 (1.32–4.69)	.005		
Proteinuria >1 g/24 hours at the time of biopsy^b^	3.19 (1.64–6.23)	.001	3.12 (1.40–6.91)	.005
Steroids weaning	1.39 (0.49–3.98)	.537		
ACEI/ARB	0.95 (0.42–2.15)	.897		
Antihypertensives (≥3)	0.83 (0.45–1.54)	.553	0.49 (0.25–0.94)	.033
**Biopsy details**				
Indication biopsy	3.76 (1.56–9.06)	.003		
C4d-positive biopsy	2.96 (1.43–6.11)	.003	2.93 (1.26–6.84)	.013
**Oxford classification MEST-C score** ^c^				
M	0.50 (0.23–1.11)	.087		
E	1.70 (0.89–3.24)	.107		
S	1.99 (1.03–3.86)	.041		
T	1.98 (1.26–3.11)	.003		
C	1.74 (0.98–3.09)	.059		
MEST-C score sum	1.25 (1.02–1.53)	.036		

ACEI: angiotensin-converting enzyme inhibitor; ARB: angiotensin receptor blocker.

^a^Univariable and multivariable associations were calculated by Cox regression. *P* < .05 was considered significant.

^b^Proteinuria at the time of biopsy was not determined in one patient.

^c^MEST-C score was not available in three patients.

A multivariable Cox model was constructed based on variables from univariable analysis. Erythrocyturia was excluded from the model since it is closely correlated with proteinuria, which was a stronger allograft loss predictor. In contrast, although there was no significant association of the antihypertensive therapy with graft failure in the univariable analysis, the multivariable model was adjusted for antihypertensive therapy, as hypertension severity is a well-known predictor of graft loss.

**Figure 3:**
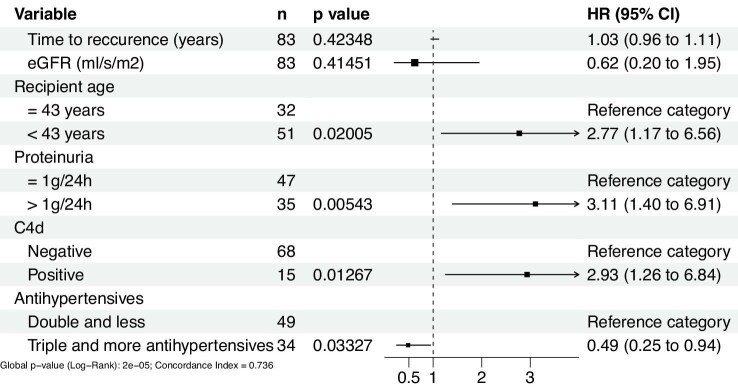
Multivariable model of allograft loss risk factors known at the time of biopsy-proven recurrence.

In the multivariable model adjusted for renal function and time to recurrence, age <43 years [HR 2.77 (95% CI 1.17–6.56), *P* = 0.02], proteinuria >1 g/day at biopsy [HR 3.12 (95% CI 1.40–6.91), *P* = .005] and C4d-positive staining [HR 2.93 (95% CI 1.26–6.83), *P* = .013] were found to be significant risk factors for allograft loss. The use of three and more antihypertensive drugs implied a moderate protective effect on allograft failure [HR 0.49 (95% CI 0.25–0.94), *P* = .033; Table 3 and Fig. 3).

### Dynamic nomogram to identify patients with recurrent IgAN at risk for premature graft loss

**Figure 4:**
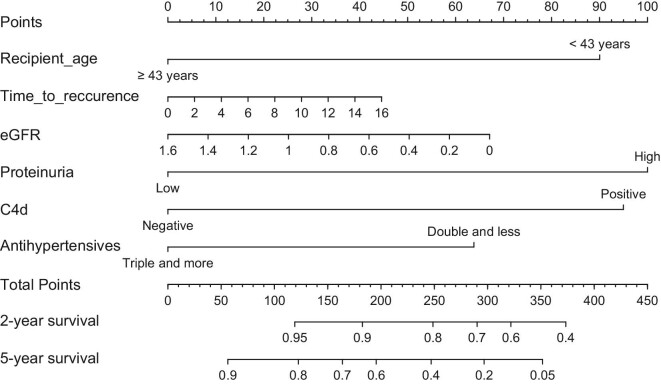
Prognostic nomogram for **(A)** 2-year and **(B)** 5-year graft survival after biopsy-proven IGAN recurrence. Time to recurrence in years; eGFR in ml/s/1.73 m^2^.

The multivariable Cox regression model based on six predictors of allograft loss after biopsy-proven IgAN recurrence (Table 3) was used to create a prognostic nomogram for 2- and 5-year survival from the biopsy (Fig. 4). To use the nomogram, the value of each predictor is read on its point scale. A total score is calculated by summing the points. In the final step, according to the sum, the corresponding predicted risk value is read on the probability scale by connecting the probability scale to the total score line. Thus the graft survival of individual patients can be calculated.

**Figure 5:**
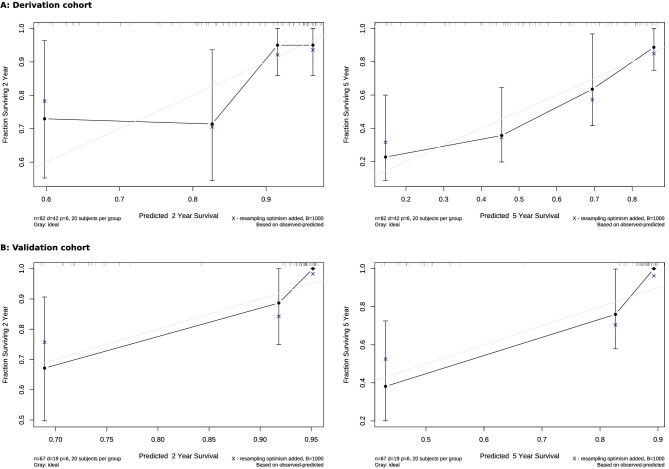
The calibration curve of the nomogram for predicting the 2- and 5-year renal survival for patients with IgAN recurrence in the **(A)** derivation and **(B)** external cohorts. The *x*-axis represents the predicted survival probability according to the nomogram and the *y*-axis displays the probability according to the Kaplan–Meier method.

The nomogram showed good accuracy in the prediction of patient outcomes. The C-index of the created nomogram was 0.736 for the derivation cohort and 0.807 for the external cohort. The nomograms were validated internally, which yielded C-indexes of 0.697 and 0.745 for the derivation and external cohorts, respectively. The calibration curves displayed good agreement between the prediction capacity of the nomograms and the actual outcomes in both the derivation and validation cohorts (Fig. 5). To facilitate the use of the nomogram in clinical practice, we created a web-based dynamic nomogram at http://research.ikem.cz/hrup/graftsurvivalreigan2021/nomogram.

## DISCUSSION

In the present study we identified younger age, female gender and retransplantation as risk factors for biopsy-proven IgAN recurrence. Next, in patients with biopsy-proven IgAN recurrence, we found younger age, proteinuria and C4d-positive histological staining to be significant variables negatively affecting allograft survival. An adjusted model for eGFR, time to recurrence and antihypertensive treatment was created to predict allograft failure after biopsy-proven recurrence with a C statistic of 0.736. This nomogram was validated on an external Norwegian cohort with C statistics of 0.807.

In native IgAN, higher proteinuria and lower eGFR were associated with poor outcome [2, 5]. Proteinuria is a well-known risk factor for the rapid progression of native kidney nephropathies as well as allograft disorders, including recurrent glomerulonephritis [25–27]. Similarly, our data suggest a negative impact of proteinuria on allograft survival. Aside from proteinuria, younger age at transplantation was associated with an increased risk for progression in our study. Similar to our results, younger age at transplantation has been described as an important risk factor for IgAN recurrence by others [1, 28, 29]. Such observations point to the severe and progressive phenotype of the original disease.

Interestingly, the use of more than three antihypertensive drugs at diagnosis of IgAN recurrence was associated with a better outcome. This observation is surprising, as hypertension itself is a well-known risk factor for progression in IgAN and other nephropathies [30]. The only possible explanation is better hypertension control in this cohort, as it is well known that a combination of various antihypertensives yields better renal outcomes [30–32].

In this study, C4d-positive histological staining of the biopsy was shown to be a strong predictor of rapid progression. Several studies have previously found complement lectin pathway activation to be associated with faster IgAN progression [7, 8, 33–36]. C4d is a well-known biomarker of complement cascade activation. C4d is associated with endocapillary proliferation [37] and positive C4d staining has been shown to be an independent risk factor for end-stage kidney disease [36]. Our study thus confirms that C4d-positive staining affects kidney allograft survival also in the case of IgAN recurrence. Recently, glomerular C4d positivity was found to double the risk of graft failure in the case of recurrence [38]. Peritubular C4d staining in kidney allografts is associated with antibody-mediated rejection (ABMR) and premature graft loss [39]. However, C4d-negative staining does not rule out an ABMR diagnosis [40], as well as the absence of donor-specific anti-HLA antibodies [41]. Our study was designed to find easily accessible variables to detect patients at the highest risk of premature graft loss and thus C4d positivity, regardless of localisation, was considered an important predictive variable.

The prognostic role of the Oxford classification MEST-C score [17] in IgAN recurrence has recently been discussed [42, 43]. Similarly, in our study we showed tubular atrophy/interstitial fibrosis and the sum of MEST-C scores to be associated with inferior kidney graft outcome in the univariable analysis. However, due to proven multicollinearity, we did not include the MEST-C score in the final model.

This study aimed to develop a publicly available prognostic nomogram based on clinical and histological variables that helps patients and caregivers estimate graft survival at the time when IgAN recurrence is histologically diagnosed. In the case of native IgAN, several predictive nomograms in IgAN were developed [44–47]. However, to the best of our knowledge, our nomogram is the first to predict outcomes in biopsy-proven IgAN recurrence. The nomogram was validated in an external cohort with similar performance, although both cohorts differed in some of the demographics.

There are also limitations that need to be discussed. Aside from the retrospective observational design, protocol biopsies were not performed later after transplantation and, similar to other studies, the rate of IgAN recurrence may be underestimated. However, case biopsies were performed in more progressive phenotypes, which improved the power, as the number of endpoints (allograft losses) was higher than in mild and non-progressive cases. Of note, recent data from the DAPA-CKD study (NCT03036150) [48] suggest gliflozins to be standard renoprotective agents in addition to RAAS blockers given to all patients with recurrent disease. Therefore, patient outcomes in the future may be better than those estimated from a nomogram constructed from retrospective data.

In conclusion, we identified a progressive phenotype of biopsy-proven IgAN recurrence. A new prognostic web-based nomogram can be used for the identification of patients at risk to whom innovative interventions should be applied.

## Supplementary Material

gfad097_Supplemental_Files

## Data Availability

The data supporting this article cannot be shared publicly due to the privacy of the individuals who participated in this study. The data will be shared upon reasonable request to the corresponding author.
