# Supplementary material for: A novel prognostic nomogram predicts premature failure of kidney allografts with IgA nephropathy recurrence
Source: Nephrol Dial Transplant. 2023 May 18;38(11):2627–36. doi: 10.1093/ndt/gfad097 (PMC10660147; doi:10.1093/ndt/gfad097)
Supplement: gfad097_Supplemental_Files [file gfad097_supplemental_files.zip › Supplementary table 1.docx]

**Table S1. Patients’ demographics in the validation cohort**

| **Validation cohort** | **n=67** |
| --- | --- |
| **Recipient’s age, years** | 35 [21,66] |
| **Time to recurrence, years** | 4,3 [0.09-40.5] |
| **eGFR** | 0.87 [0.2, 1.7] |
| **Antihypertensives - 3 or more** | 14 (21%) |
| **C4d + in biopsy** | 1 (1.5%) |
| **ERY>10/µl at the time of biopsy** | 47 (70%) |
| **PU >1g/24h at time of biopsy** | 27 (40%) |

Values are presented as Number (%) or Median [min, max]. eGFR, estimated glomerular filtration rate; ERY, erythrocyturia; PU, proteinuria
