# Supplementary material for: A novel prognostic nomogram predicts premature failure of kidney allografts with IgA nephropathy recurrence
Source: Nephrol Dial Transplant. 2023 May 18;38(11):2627–36. doi: 10.1093/ndt/gfad097 (PMC10660147; doi:10.1093/ndt/gfad097)
Supplement: gfad097_Supplemental_Files [file gfad097_supplemental_files.zip › Supplementary table 2.docx]

**Table S2. Risk factors of recurrence in patients with IgA nephropathy in the first kidney transplant (66 patients with reIGAN out of 376)**

| **Patients after 1st transplantation** | | | | |
| --- | --- | --- | --- | --- |
| **Predictors** | **Univariable analysis^a^**  **HR (95%CI)** | **P value** | **Multivariable analysis^a^**  **HR (95%CI)** | **P value** |
| Recipient age, <43 years | 2.49 (1.48- 4.19) | <0.001 | 2.49 (1.47- 4.24) | 0.001 |
| Peak PRA^b^ | 1.00 (0.99- 1.01) | 0.77 |  |  |
| HLA mismatch^c^ | 1.08 (0.88- 1.32) | 0.442 |  |  |
| Recipient gender, female | 1.78 (1.04- 3.05) | 0.036 | 1.98 (1.14- 3.46) | 0.016 |
| Dialysis vintage, months^d^ | 1.00 (0.99- 1.01) | 0.93 |  |  |
| Cold ischaemia, hours^e^ | 0.98 (0.95- 1.02) | 0.300 |  |  |
| Donor age, years^f^ | 1.02 (1.00- 1.04) | 0.044 | 1.01 (0.99-1.04) | 0.077 |
| Donor gender, female^f^ | 1.71 (1.02- 2.89) | 0.042 | 1.63 (0.96-2.75) | 0.068 |
| Type of donor, living | 1.19 (0.70-2.04) | 0.508 |  |  |

HR, hazard ratio; CI, confidence interval; PRA, panel reactive antibody; HLA, human leukocyte antigen.

^a^ Univariable and multivariable associations were calculated by Cox regression, results are expressed with hazard ratios and their 95% confidence intervals, p < 0.05 for significance

^b^ PRA measurement not available for 1 patient

^c^ HLA mismatch not available in 26 patients

^d^ Dialysis vintage not known in 43 patients

^e^ Cold ischaemia not known in 50 patients

^f^ Donor age and gender not known in 20 and 19 patients, respectively
